# *De Novo* Assembly, Gene Annotation and Marker Development Using Illumina Paired-End Transcriptome Sequences in Celery *(Apium graveolens* L.)

**DOI:** 10.1371/journal.pone.0057686

**Published:** 2013-02-28

**Authors:** Nan Fu, Qian Wang, Huo-Lin Shen

**Affiliations:** College of Agriculture and Biotechnology, China Agricultural University, Beijing, China; Yale School of Medicine, United States of America

## Abstract

**Background:**

Celery is an increasing popular vegetable species, but limited transcriptome and genomic data hinder the research to it. In addition, a lack of celery molecular markers limits the process of molecular genetic breeding. High-throughput transcriptome sequencing is an efficient method to generate a large transcriptome sequence dataset for gene discovery, molecular marker development and marker-assisted selection breeding.

**Principal Findings:**

Celery transcriptomes from four tissues were sequenced using Illumina paired-end sequencing technology. *De novo* assembling was performed to generate a collection of 42,280 unigenes (average length of 502.6 bp) that represent the first transcriptome of the species. 78.43% and 48.93% of the unigenes had significant similarity with proteins in the National Center for Biotechnology Information (NCBI) non-redundant protein database (Nr) and Swiss-Prot database respectively, and 10,473 (24.77%) unigenes were assigned to Clusters of Orthologous Groups (COG). 21,126 (49.97%) unigenes harboring Interpro domains were annotated, in which 15,409 (36.45%) were assigned to Gene Ontology(GO) categories. Additionally, 7,478 unigenes were mapped onto 228 pathways using the Kyoto Encyclopedia of Genes and Genomes Pathway database (KEGG). Large numbers of simple sequence repeats (SSRs) were indentified, and then the rate of successful amplication and polymorphism were investigated among 31 celery accessions.

**Conclusions:**

This study demonstrates the feasibility of generating a large scale of sequence information by Illumina paired-end sequencing and efficient assembling. Our results provide a valuable resource for celery research. The developed molecular markers are the foundation of further genetic linkage analysis and gene localization, and they will be essential to accelerate the process of breeding.

## Introduction

*Apium graveolens* L. (family Apiaceae), originated from the Mediterranean basin, is a biennial species grown worldwide. There are three distinct taxonomic varieties, var. *dulce* (celery), var. *rapaceum* (celeriac), and var. *secalinum* (smallage) [1]. Celery is the most widely grown variety and it was spread to China in the Han Dynasty (the second century B. C.). After a long period of domestication, cultivars with slender petiole known as Chinese celery or local celery came into being [2]. Celery is rich in a variety of nutrients like vitamins, minerals and proteins and it has many pharmacological efficacies, which make it an increasing popular vegetable to consumers [3], [4]. In China, celery growing area has been increased from 0.54 million hectare in 2004 to 0.57 million hectare in 2006 according to the data statistics, almost accounting for 3% of the total vegetable planting area throughout the country. So it is essential to breed new celery varieties and enrich commercial species.

Molecular marker assisted selection has become a more and more important auxiliary means for conventional breeding. However, only a few molecular markers have been indentified in celery. After the development of isozyme markers and morphological markers [5], [6], Huestis et al [7] initially developed 21 restriction fragment-length polymorphism (RFLP) markers. Yang and Quiros [8] first used random amplified polymorphic DNA (RAPD) markers in celery to distinguish different cultivars. Domblides [9] did the similar research using RAPD markers. Then amplified fragment length polymorphism (AFLP) markers were used in celery for cultivar classification and genetic diversity analysis [10], [11]. In recent decades, simple sequence repeats (SSRs) as a marker system have been widely used. However, the lack of expressed sequence tags (ESTs) and genomic sequences prevents the development of SSR markers in celery. So far only 11 SSR markers have been reported [12]. Wang et al [13] used the 11 SSR makers and 60 sequence-related amplified polymorphism (SRAP) primer combinations to study the genetic diversity of 68 celery accessions.

During the last decades, large amounts of transcriptomic and genomic sequences have been available in many model organisms. For *Apium graveolens*, only about 2,000 ESTs were deposited in GenBank database. Therefore, extensive genomic or transcriptomic sequences are badly needed for *Apium graveolens*, which can be used for new genes discovery, molecular markers development, gene localization, and comparative genomics and so on. Given that the celery is a diploid with a large genome (3×10^9 ^bp) and a high degree of heterozygosity, it is infeasible to consider whole genome sequencing because of the high costs and time-consuming. Fortunately, the advent of transcriptome sequencing provides an alternative to the whole genome sequencing. Transcriptome sequences exclude non-coding DNA, thus the sequences obtained must contain a high content of functional information [14], [15] and are beneficial to reveal the molecular mechanism of functional genes [16].

A number of next-generation sequencing (NGS) technologies such as Roche/454, Illumina and AB SOLiD have recently become available. These technologies are capable of generating high-throughput reads at a relatively low cost and are being utilized for research in many areas like *de novo* sequencing, genome re-sequencing and whole genome or transcriptome analysis. In the past few years, the 454 pyrosequencing has been preferred for non-model organisms [17]–[20] due to its longer read length. With shorter read length, Illumina and SOLID platforms were confined to re-sequencing applications which rely on a reference sequence. Recently, with increased read length by Illumina technology improvement and development of new computational tools, short reads can be assembled and used for transcriptome analysis. *De novo* assemblies using sequence reads from Illumina technology have been reported in many non-model organisms without a reference sequence [21]–[23]. NGS technologies prove to be efficient, inexpensive and reliable for genome and transcriptome sequencing, and suitable for non-model organisms such as celery.

In this study, we sampled from different celery tissues (roots, stems, petioles and leaves) and used Illumina paired-end sequencing technology to generate a large-scale EST dataset and develop a set of EST-SSRs. This study is the first try to characterize the complete transcriptome of celery by analyzing large-scale transcriptome sequences. These sequences will serve as a valuable resource for novel gene discovery, development of molecular markers, gene mapping and comparative genomics.

## Materials and Methods

### Plant Material

Celery line “Q 111” was grown at the experimental station of China Agricultural University, Beijing, China. Samples were collected from roots, stems, petioles and leaves, frozen immediately in liquid nitrogen and stored at −80°C until use.

### RNA Extraction and Library Preparation for Transcriptome Analysis

Total RNA was extracted using the Trizol reagent according to the manufacture’s instructions (Invitrogen). The total RNA concentration was quantified using UV spectrophotometry, and quality of total RNA is checked by electrophoresis in 1% agarose gel. Equal volumes of RNA from each of the four tissues were pooled. Paired-end libraries with approximate average insert lengths of 200 base pairs were synthesized using the Genomic Sample Prep kit (Illumina, San Diego, CA) according to manufacturer’s instructions. Prior to cluster generation, library concentration and size were assayed using the Agilent DNA1000 kit (Agilent, USA) on a 2100 Bioanalyzer (Agilent, USA). Libraries were sequenced as 100-mer×2 on a Hi-Seq 2000 equipped with a paired-end module at the MininGene Biotechnology Co. Ltd (Beijing, China).

### Illumina Reads Processing and Assembly

A Perl script was written to remove low quality sequences (reads with a base quality less than 20). Then the high quality reads were assembled by Trinity [24] with default settings except K-mer value to construct unique consensus sequences.

### Gene Annotations and Classifications

Functional annotations were performed by sequence comparison with public databases. All unigenes were compared with the NCBI non-redundant protein database (http://www.ncbi.nlm.nih.gov/), Swiss-Prot database (http://www.expasy.ch/sprot), the Clusters of Orthologous Groups database (http://www.ncbi.nlm.nih.gov/COG/) using BLASTx [25] with an E-value of less than 1e^−5^, 1e^−10^ and 1e^−10^ respectively. InterPro domains [26] were annotated by InterProScan [27] Release 16.0 and functional assignments were mapped onto Gene Ontology (GO) (http://www.geneontology.org/). Then we made GO classification and drew GO tree using WEGO (http://wego.genomics.org.cn/cgi-bin/wego/index.pl). Pathway assignments were carried out based on the KEGG database (http://www.genome.jp/kegg). Unigenes were first compared with KEGG database using BLASTx with an E-value of less than 1e^−10^, and then a Perl script was developed to retrieve KO (KEGG Orthology) information from BLASTx results and we established pathway correlation between unigenes and database.

### EST-SSR Detection and Primer Design

Potential SSR markers were detected among the 42,280 unigenes using the MISA tool [28] (http://pjrc.ipk-gatersleben.de/misa/). We searched for SSRs with motifs ranging from mono- to hexa-nucleotides in size. The minimum of repeat units were set as follows: ten repeat units for mono-nucleotide, six for di-nucleotides and five for tri-, tetra-, penta- and hexa-nucelotides. Mono-nucleotide repeats were discarded given that it was difficult to distinguish genuine mono-nucleotide repeats from polyadenylation products and some mono-nucleotide repeats were generated by base mismatch or sequencing errors. Primer pairs were designed using Primer3 (http://fokker.wi.mit.edu/primer3/) with default parameters and 80 randomly selected primer pairs (Table S1) were synthesized (Beijing Sunbiotech co., Ltd) for polymorphism detection in celery.

### Survey of EST-SSR Polymorphism

Polymorphism was validated among 31 celery accessions (Table S2). Genomic DNA was extracted from celery tender leaves using a modified version of the cetyltrimethyl ammonium bromide (CTAB) method [29]. PCR amplifications were conducted in a final volume of 10 µL containing 3.5 µL 2×Taq PCR MasterMix (Beijing Biomed co., Ltd), 4.5 µLddH2O, 0.5 µL of each primer (5 µM) and 1 µL of template (aprox. 20 ng/µL). PCR was performed as follows: denaturation at 94°C for 5 min, followed by 38 cycles of 30s at 94°C, 30s at Tm (annealing temperature), 1 min at 72°C and a final step at 72°C for 10 min. PCR products were firstly detected by agarose gel elctrophoresis and the products possessing single band or only a few bands were subjected to 7% polyacrylamide gel to separate alleles. With regard to those had no bands or multiple bands, we optimized the PCR condition to get better products for separation of alleles. PCR products were mixed with a volume of loading buffer and then denatured at 95°C for 10 min before being loaded on the polyacrylamide gel.

### Data Collection and Cluster Analysis

The presence or absence of each single band was coded by 1 or 0 respectively. Scored data from polymorphic loci were used to calculate the polymorphism information content (PIC) according to the formula of PIC = 1–∑pi^2^ (pi is the frequency of i^th^ allele for each locus) [30]. Observed heterozygosity (*Ho*) and expected heterozygosity (*He*) were calculated using the Popgene software version 1.31 [31]. A genetic similarity matrix was constructed using the NTSYSpc 2.1 software [32]. UPGMA (un-weighted pair group method with arithmetic mean) cluster analysis was performed to develop a dendrogram.

## Results

### Illumina Paired-end Sequencing and de novo Assembly

**Figure 1:**
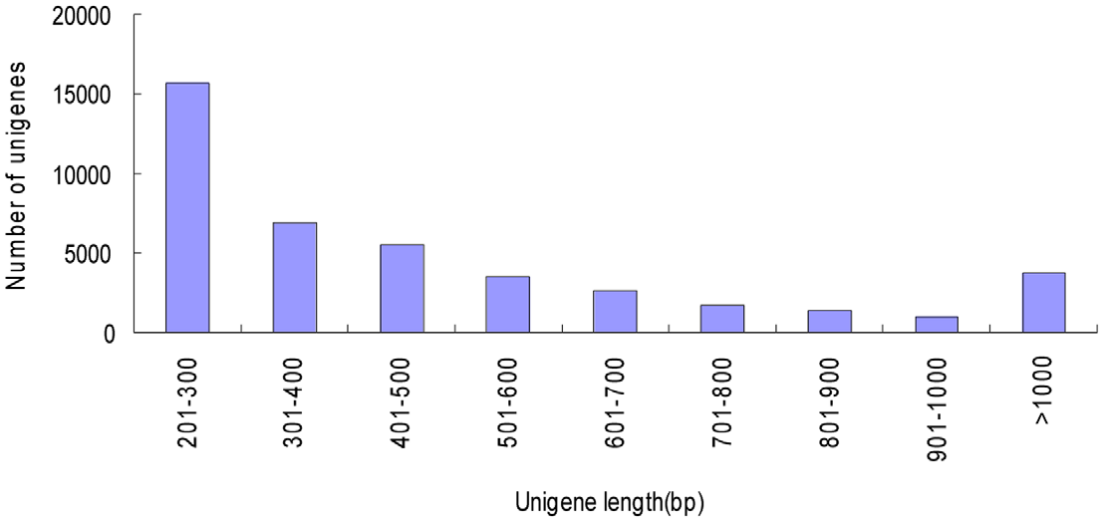
Length distribution of the celery unigenes *de novo* assembled from 42280 ESTs.

The paired-end sequencing yielded 2×101-bp reads from either end of the cDNA fragment. In this study, a total of 27,154,728 raw sequencing reads were generated from a 200 bp insert library, encompassing 2.7 Gb of sequence data. After stringent quality assessment and data filtering, reads with Q20 bases (those with a base quality greater than 20) were selected as high quality reads for further analysis. 25,915,104 (89.33%) reads were deemed high quality reads, of which 621,320 (2.4%) were ribosomal. The sequences have been deposited in DDBJ Sequence Read Archive (DRA, http://trace.ddbj.nig.ac.jp/dra/) with accession number DRA000903. We used the Trinity method with optimized k-mer length of 31 for *de novo* assembly. Finally all short sequences were assembled into 42,280 unigenes with an average length of 502.6 bp and a median length of 604 bp. There were 22,262,653 (85.91%) reads assembled into transcripts. The number of reads aligned to each unigene ranges form 1 to 92,345, with an average number of 228. The majority of the reads were in the range of 200–400 bp, which accounted for 53.46%. There were 15,906 unigenes (37.62%) in the length range of 401 to 1000 bp and 3,769 unigenes (8.91%) with length more than 1000 bp (Figure 1).

### Annotation of all Non-redundant Unigenes

**Figure 2:**
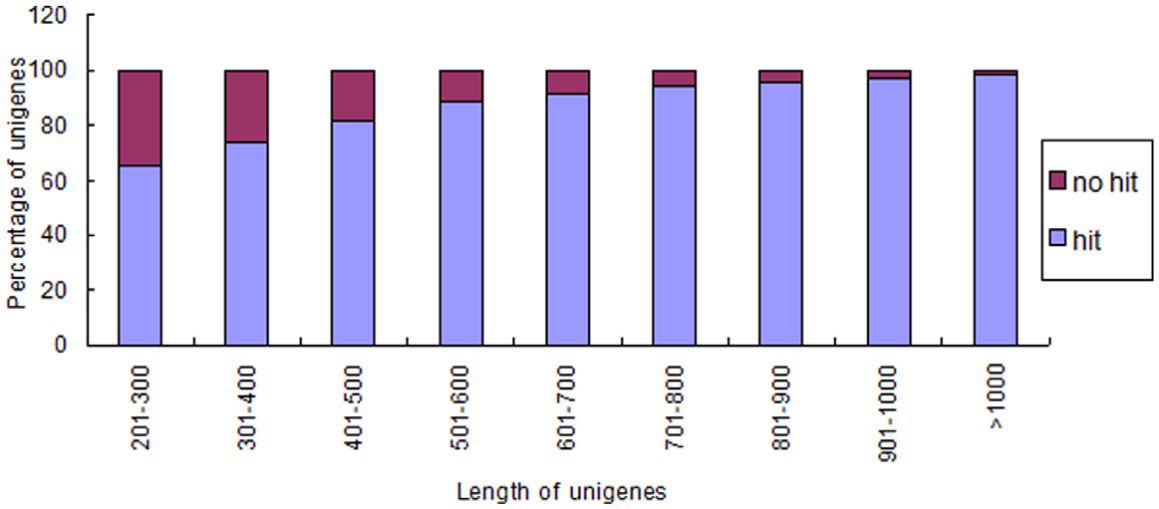
Comparison of unigene length between hit and no hit unigenes. Longer contigs were more likely to have BLASTx homologs in protein database.

**Figure 3:**
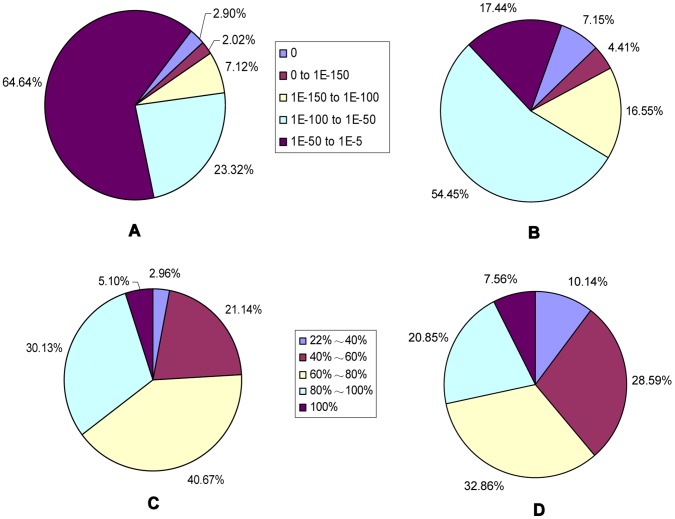
Characteristics of similarity search of unigenes against Nr and Swiss-Prot databases. (A) E-value distribution of BLAST hits for each unigene with a cutoff E-value of 1E-5 in the Nr database. (B) E-value distribution of BLAST hits for each unigene with a cutoff E-value of 1E-5 in the Swiss-Prot database. (C) Similarity distribution of the top BLAST hits for each unigene in Nr database. (D) Similarity distribution of the top BLAST hits for each unigene in Swiss-Prot database.

A sequence similarity search was conducted against the Nr database (E-value<1e^−5^) and Swiss-Prot database (E-value<1e^−10^) using the BLASTx algorithm, with the outcome that 33,160 (78.43%) and 20,686 (48.93%) unigenes showed homology with sequences in the Nr and Swiss-Prot database respectively. Our results showed that more than 80% of unigenes over 400 bp in length had BLAST matches against Nr database, whereas only 65.34% of unigenes shorter than 300 bp did (Figure 2). The same tendency was observed in the BLAST against Swiss-Prot database. We also made analysis of E-value and similarity distributions of the top hits in the Nr database. There were 35.92% and 35.49% of the sequences showing significant homology (E-value<1e^−50^) and high similarity (greater than 80%), respectively (Figure 3A and 3C). For E-value and similarity distributions of the top hits in the Swiss-Prot database, the percentages were 32.87% and 28.71% (Figure 3B and 3D).

The celery unigenes were also compared with all the 25,276 Apiaceae ESTs in NCBI and 58,751 carrot transcriptome sequences [33] with the E-value of less than 1e^−10^, and with the result that 19,878 (47.02%) and 2,886 (6.83%) unigenes had matches against carrot transcripts and Apiaceae ESTs, respectively. Similarly, 19,872 carrot transcripts and 2,881 Apiaceae ESTs had significant matches to the celery transcripts when they were respectively compared to the celery transcripts.

In addition, we made an ORF prediction analysis. ORF was predicted using getORF from EMBOSS package and most unigenes (86.94%) were identified to have an ORF (Table S3). Among those with ORF, 31,352 (85.30%) unigenes had annotations against at least one of the following databases: Nr, SwissProt and KEGG database. They are probably genes encoding specific proteins, while unigenes without hits (14.70%) may represent new genes or errors in the assembly procedure.

### Functional Classification by GO

**Figure 4:**
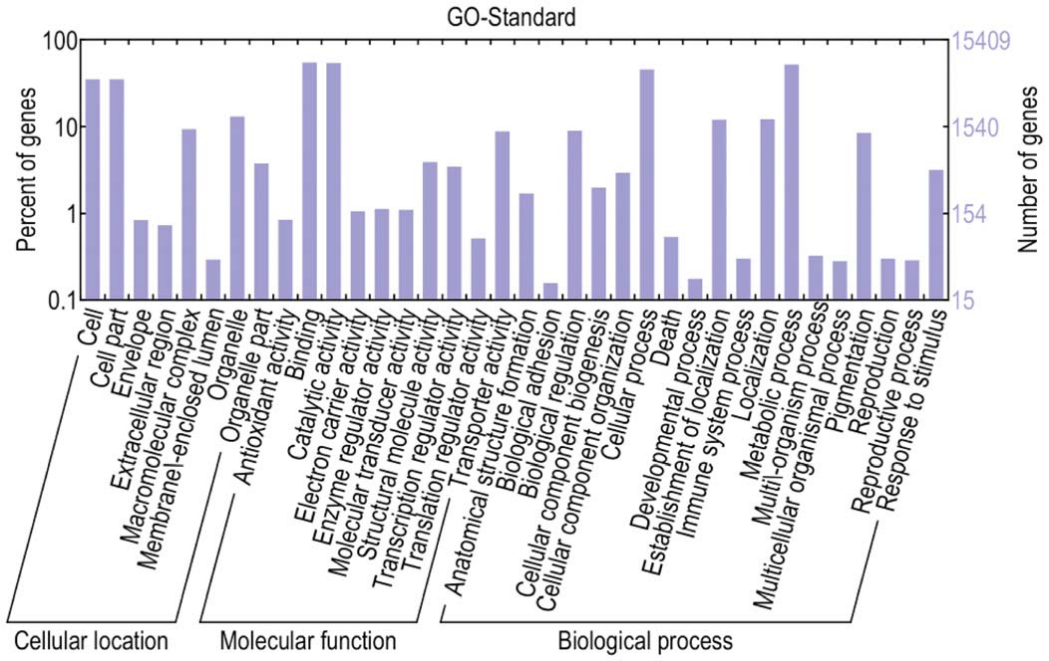
Gene Ontology classifications of assembled unigenes. The unigenes are summarized into three main categories: biological process, cellular location, and molecular function. In total, 15,409 unigenes with BLASTx matches were assigned to gene ontologies.

Gene Ontology (GO) provides ontologies of defined terms representing gene product properties, and it has ontologies that describe gene products in terms of their associated biological processes, cellular components and molecular functions. In this study, 15,409 unigenes could be assigned to one or more ontologies and we assigned each unigene to a set of GO Slims. A summary with unigenes classified to each GO slim term is shown in Figure 4. Totally, 5,535 unigenes were grouped under cellular components, 13,934 unigenes under molecular functions and 11,210 unigenes under biological processes. Metabolic process (7,908 unigenes, 70.54%) and cellular process (7,024 unigenes, 62.66%) were the most highly represented groups under the biological process category. Genes involved in other important biological processes such as biological regulation, stimulus response, reproduction and developmental process were also identified. Furthermore, we also found that a relatively large numbers of unigenes (11.57%) were involved in the metabolism of pigmentation, which may play a role in the petiole color formation. For the cellular components category, cell and cell part were the most highly represented groups. Regarding molecular functions, binding and catalytic activity represented the majorities of the category with a large number of ligases, transferases, hydrolases, oxidoreductases annotated.

### Functional Classification by COG

**Figure 5:**
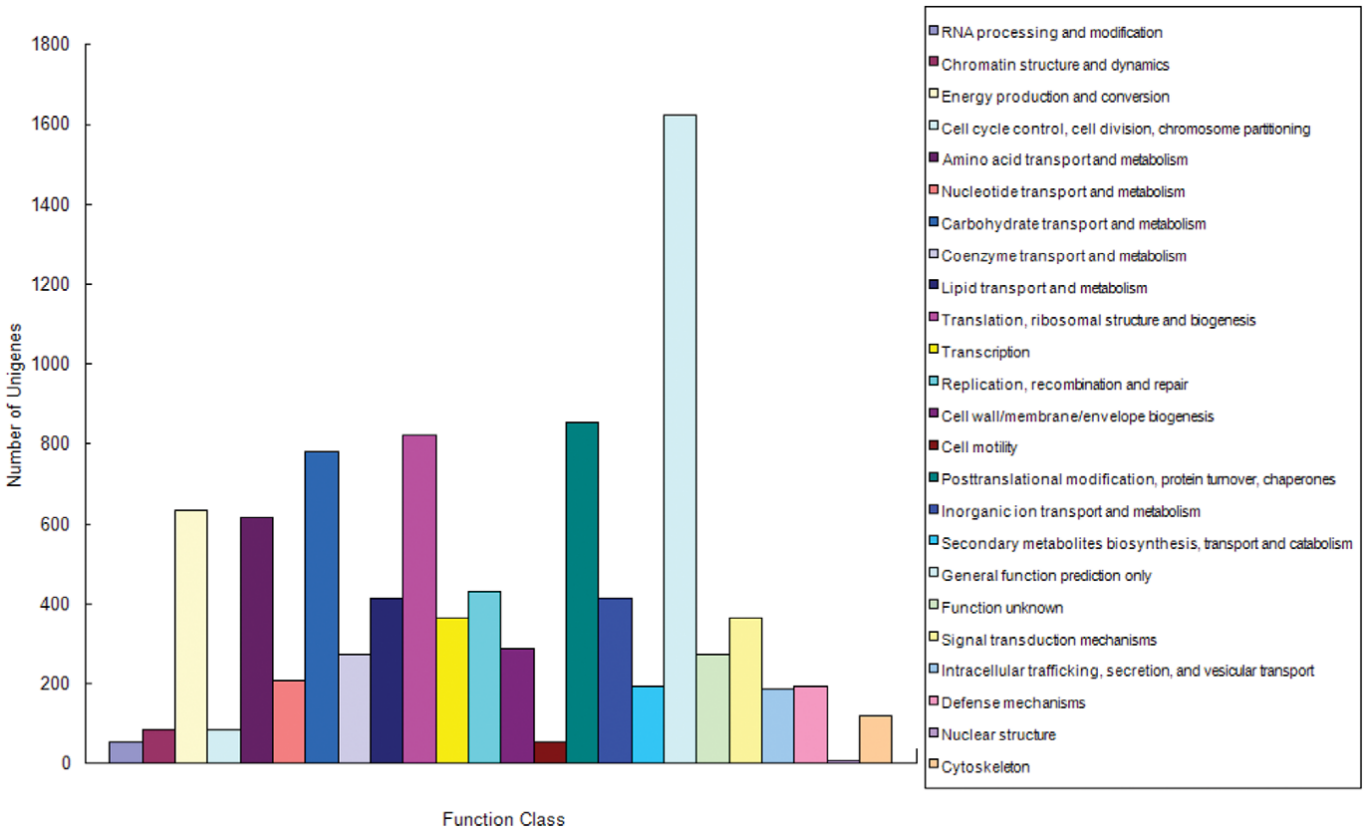
Clusters of orthologous groups (COG) classification. In total, 10,473 sequences were grouped into 24 COG classifications.

All unigenes were subjected to a search against the COG database for functional prediction and classification. 9,332 sequences were assigned to COG classifications (Figure 5). Among the 24 COG categories, the cluster for general function prediction only (17.38%) was the largest group, followed by posttranslational modification, protein turnover and chaperones (9.14%), translation, ribosomal structure and biogenesis (8.82%) and carbohydrate transport and metabolism (8.36%).

### Functional Classification by KEGG

**Figure 6:**
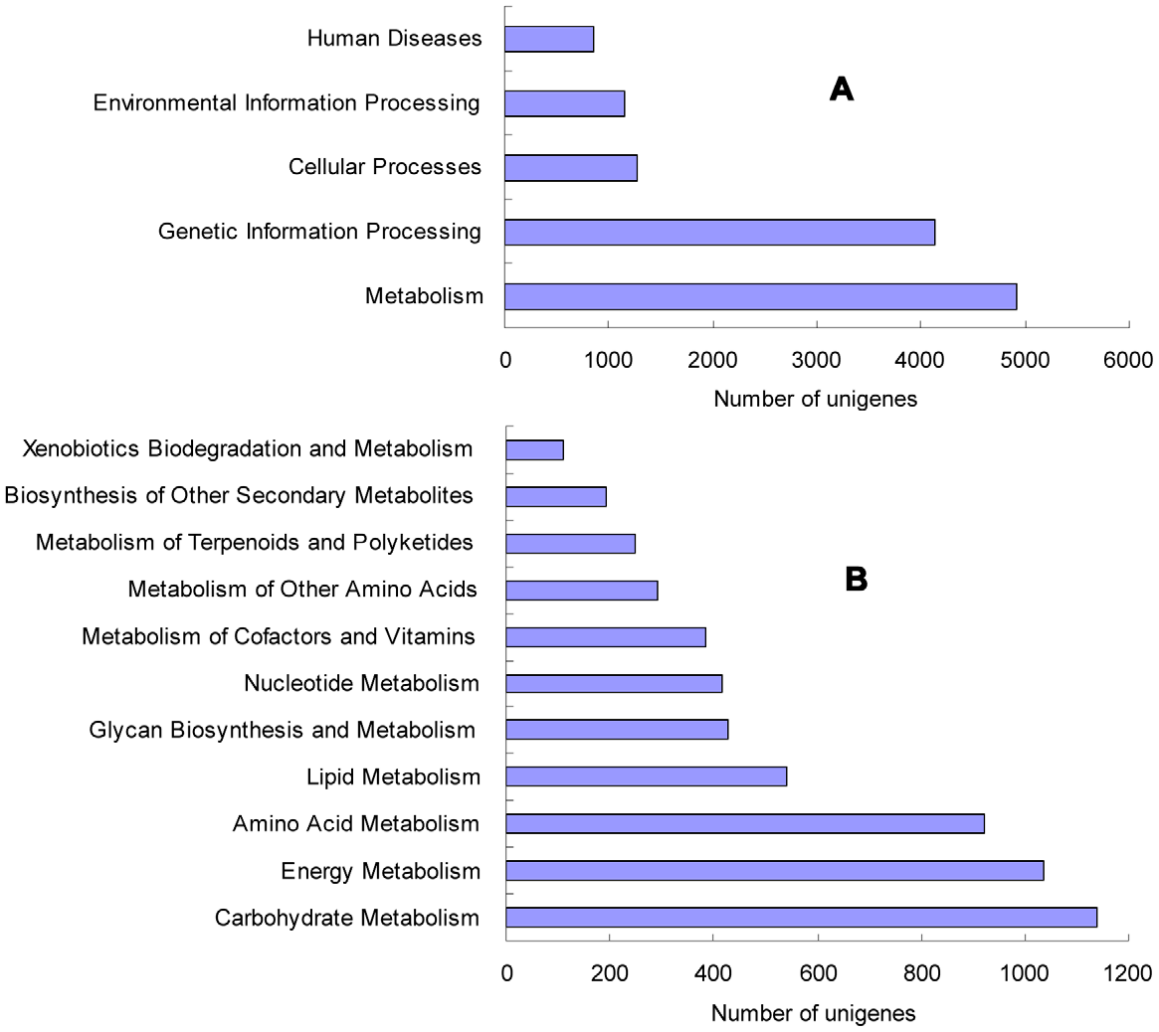
Pathway assignment based on KEGG. (A) Classification based on metabolism categories; (B) Categories classified by KEGG.

Unigenes were compared with KEGG using BLASTx with an E-value of less than 1e^−10^ and the corresponding pathways were established. Out of the 42,280 unigenes, 30,704 (72.62%) were annotated and 9,982 (32.51%) were assigned to KEGG pathways. Among the 9,982 unigenes, 4,921 were assigned to the metabolic pathways, composing the largest group of the five categories classified by KEGG (Figure 6B). The KEGG metabolic pathways (Figure 6A) mainly contained carbohydrate metabolism, energy metabolism, amino acid metabolism, lipid metabolism and glycan biosynthesis and nucleotide metabolism. In addition to the unigenes assigned to the metabolism pathways, 4,128 unigenes were sorted to the genetic information processing involving folding, sorting, degradation, replication and repair, transcription and translation. Additionally, there were 1,265 and 1,149 unigenes classified to cellular processes and environmental information processing respectively.

### Genes Potentially Controlling Petiole Traits and Bolting

There exist green and yellow petioles in celery, and green petiole color has been reported to be dominant to yellow [34], [35]. Yellowing mutation is a common phenomenon in the nature and the celery with yellow petiole is considered as a mutant of the celery with green petiole. Similar phenomenons have been reported in organism like cucumber [36]–[38], pepper [39] and carrot [40], [41]. Yellowing mutation plays an important role in studies of plant photosynthesis mechanism, Chlorophyll biosynthetic pathway, genetic control mechanism and genetic breeding. It has been reported that the content of chlorophyll in yellowing plants is dramatically lower than that in normal plants [34], [35], [36], [38], [41]. The celery unigenes contain genes potentially controlling petiole color (Table S4). And we found 85 unigenes participated in chlorophyll metabolism pathways. Combined with the GO annotations, there were 8 unigenes involved in chlorophyll biosynthetic process and 4 in chlorophyll catabolic process, and annotations of these unigenes were consistent with Nr or Swiss-Prot database.

According to petiole organizational structure, celery can be divided into hollow and solid types. Collenchyma, presented underneath the petiole epidermis, is the main mechanical organization of celery petiole with the function of supporting the whole plant. Cellulose and pectin are the main components of collenchyma cells walls which are uneven thickened, and the both may be correlated with the formation of hollow or solid petioles. About 100 homologs involved in metabolism of cellulose and pectin were found in our transcripts (Table S5).

Early bolting, being a great obstacle in production, dramatically affects the commercial quality of celery. The unigenes include orthologs of genes involved in flowering (Table S6) such as *ELF8* (*EARLY FLOWERING 8*), *ELF7* (*EARLY FLOWERING 7*), *FCA* (*flowering time control*), *FLC* (*FLOWERING LOCUS C*) [42], *RKF3* (*RECEPTOR-LIKE KINASE IN FLOWERS 3*) [43], *TM6* and *AP3* (*flowering-related B-class MADS-box protein*) [44], *TFL2* (*TERMINAL FLOWER 2*) [45] and *PIE1* (*PHOTOPERIOD-INDEPENDENT EARLY FLOWERING 1*) [46]. In addition, the date of bolting and flowering can be regulated by plant hormones and environmental conditions and it has been reported that gibberellins (GA) can facilitates date of bolting [47], [48]. We detected some orthologs of genes including gibberellin 2-oxidase (*GA2ox1, GA2ox2*) and gibberellin receptor *GID1* which were involved in signal transduction pathways [49].

### EST-SSR Discovery: Distribution and Frequencies

**Figure 7:**
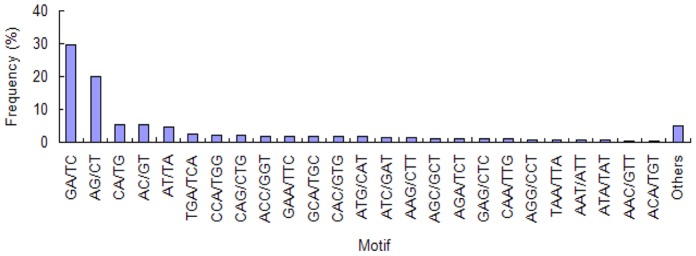
Frequency distribution of SSRs based on motif types. The GA/TC di-nucleotide repeat motif was the most abundant motif detected.

All of the 42,280 unigenes generated in this study were used to mine potential microsatellites using MISA software. A total of 2,997 SSRs were identified in 2,601 unigenes. Of all the SSR containing unigenes, 339 sequences contained more than one SSR and 472 SSRs were present in compound form (Table 1). And we found 1 SSR per 10 Kb on average in this study, which was significantly different from the frequency in coffee (1 SSR per 1.5 Kb) [50] and in cotton (1 SSR per 20 Kb) [51]. Among the 2,997 SSRs identified, the di-nucleotide repeat motifs were the most abundant types (43.98%), followed by mono- (32.93%), tri- (21.62%), tetra- (0.97%), hexa- (0.13%) and penta-nucleotide (0.37%) repeat motifs. The frequencies of EST-SSRs with different numbers of tandem repeats were summarized in Table 2. SSRs with six tandem repeats (22.62%) were the most common, followed by ten tandem repeats (18.75%), five tandem repeats (12.98%), and seven tandem repeats (12.95%). Given that the mono-nucleotide repeats may not be accurate because of the sequencing errors and assembly mistakes, mono-nucleotide repeats were not used for further analysis. The most common type of di-nucleotide was GA/TC which accounted for 29.55% of the repeats, followed by AG/CT (20.15%) and CA/TG (5.57%). Among the tri-nucleotide repeats, the TGA/TCA (2.74%) and CCA/TGG (2.39%) were the most frequent motifs (Figure 7).

**Table 1:** Summary of SSR searching results.

Item	Number
Total number of unigenes examined	42,280
Total size of examined unigenes (bp)	21,250,238
Number of SSR-containing unigenes	2,578
Total number of identified SSRs	2,997
Avarage number of SSRs per 1 K	0.1
Number of unigenes containing 1 SSR	2,239
Number of unigenes containing more than 1 SSR	339
Number of SSRs present in compound formation	478

**Table 2:** Distribution of identified SSRs using the MISA software.

Motif	Repeat Numbers								total	%
	5	6	7	8	9	10	11	>11		
Mono-	0	0	0	0	0	451	194	342	987	32.93
Di-	0	514	284	213	128	110	66	3	1,318	43.98
Tri-	356	157	102	25	5	0	0	3	648	21.62
Tetra-	23	4	2	0	0	0	0	0	29	0.97
Penta-	1	2	0	0	0	1	0	0	4	0.13
Hexa-	9	1	0	0	1	0	0	0	11	0.37
Total	389	678	388	238	134	562	260	348	2,997	100
%	12.98	22.62	12.95	7.94	4.47	18.75	8.68	11.61%	100	

### Polymorphism Test of SSR Markers

31 celery accessions were used to evaluate the rate of amplication and polymorphism using 80 primer pairs randomly selected, of which 65 pairs (81.25%) successfully amplified fragments and 47 pairs (72.31%) produced PCR amplicons at the expected size. We also found 28 microsatellite loci showed allelic polymorphism (Table 3). The number of alleles per locus raged from two to four with an average of 2.68. The value of observed heterozygosity (*Ho*) varied from 0 to 0.92 (mean: 0.14), while the expected heterozygosity (*He*) varied form 0.06 to 0.74 (mean: 0.36). Polymorphism information content (PIC) values ranged form 0.06 to 0.72 with an average of 0.35.

**Table 3:** Characteristics of the primer pairs possessing polymorphic SSR loci texted in 31 different accessions of *A. graveolens*.

Primer	SSRs	No.of alleles	*Ho*	*He*	PIC
QC10	(GAAA)5	3	0.00	0.12	0.12
QC 12	(AACT)5	2	0.00	0.16	0.15
QC 24	(CT)6	2	0.34	0.46	0.45
QC 28	(GAG)8	4	0.06	0.56	0.55
QC 34	(ATT)5	2	0.09	0.24	0.24
QC 41	(TC)6	4	0.31	0.74	0.72
QC 43	(AT)6	3	0.03	0.15	0.15
QC 46	(TG)6	3	0.41	0.54	0.53
QC 47	(AG)6	3	0.10	0.55	0.54
QC 48	(TC)6	2	0.13	0.12	0.12
QC 49	(AG)6	2	0.00	0.18	0.17
QC 51	(TTG)6	3	0.92	0.58	0.57
QC 52	(GCA)5	4	0.00	0.59	0.58
QC 53	(GCT)6	3	0.03	0.51	0.51
QC 55	(TCA)5	3	0.03	0.56	0.55
QC 56-1	(ACC)6	2	0.00	0.18	0.18
QC 56-2	(ACC)6	3	0.04	0.23	0.23
QC 57	(TCA)5	2	0.00	0.06	0.06
QC 62	(TGA)6	2	0.00	0.06	0.06
QC 63	(GTG)6	2	0.20	0.50	0.49
QC 67	(GGT)7	2	0.00	0.06	0.06
QC 70	(TCA)5	4	0.11	0.63	0.60
QC 71	(GGT)7	3	0.03	0.19	0.18
QC 72	(TCA)7	2	0.14	0.38	0.38
QC 73	(CAG)6	2	0.00	0.06	0.06
QC 75	(CAG)5	2	0.86	0.50	0.49
QC 80	(GT)6	3	0.07	0.52	0.51
QC 86	(AT)8	3	0.03	0.54	0.53
Mean		2.68	0.14	0.36	0.35

*Ho*: Observed heterozygosity; *He*: Expected heterozygosity; PIC: Polymorphism Information Content.

**Figure 8:**
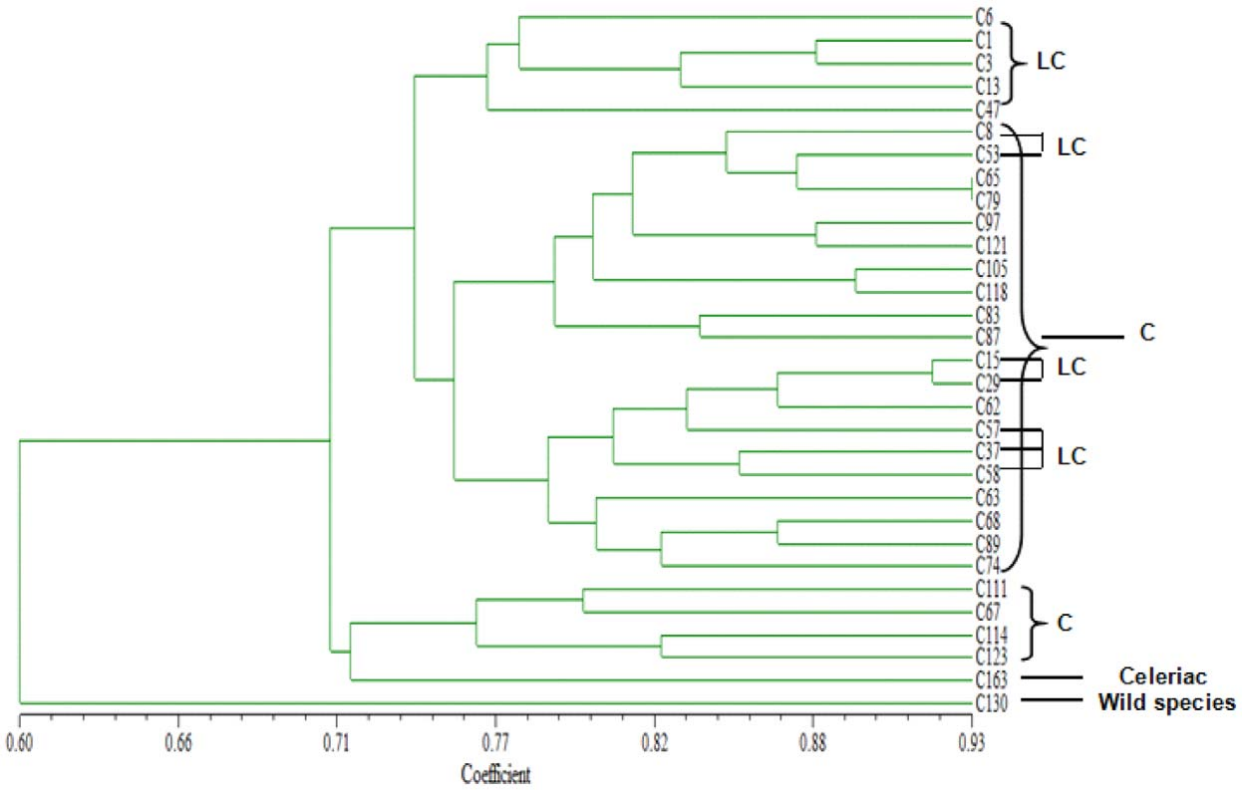
Similarity relationships of 31 different accessions of *A. graveolens* based on 28 EST-SSR loci. LC: local celery; C: celery.

The polymorphic SSR markers were then used to perform genetic correlation analysis among the 31 different *A. graveolens* accessions, and dendrograms were constructed from the genetic similarity matrix (Figure 8). At the genetic distance of 0.6, cultivated and wild species were separated. Within the cultivated species, almost all lines of *A. graveolens* L. var. *dulce* formed a cluster, except that five lines (C111, C67, C114, C123, and C163) were classified into a group with *A. graveolens* L. var.*rapaceum* (C163). The lines belonging to *A. graveolens L.* var. *dulce* were further classified into local celery (cultivars developed through ancient introductions) and celery (modern cultivars introduced from Europe and America). And most local celery formed one group with the remaining scattered in the celery group.

## Discussion

### Illumina Paired-end Sequencing and Assembly

With the introduction of next-generation sequencing, transcriptome sequencing, which displays a great advantage over traditional Sanger sequencing by lowering the cost, increasing the throughput and eliminating complicated bacterial cloning, has become an important tool used in research. Roche GS FLX platform, due to its longer read length, has been widely used for *de novo* transcriptome sequencing in many organisms like pigeonpea [52], yellow lupin [53], lodgepole pine [18], [54] and so on. Illumina was initially limited to organisms with reference genomes available due to its shorter read length [55]–[58]. While in recent years, with the improvement of read length by paired-end sequencing and the development of bioinformatics and computational methods [24], [59]–[62], the relatively short reads can be effectively assembled and has been successfully used for non-model organisms [63]–[65]. In accordance with the previous reports, our results also proved that short reads from Illumina paired-end sequencing could be well assembled and used for transcriptome analysis, gene identification and marker development in celery. Here, approximately 13 million paired-end reads were generated from HiSeq 2000 and they were finally assembled to 42,280 unigenes with an average length of 502.6 bp. The unigenes were larger than that reported for *Lycoris sprengeri* and cauliflower transcriptomes using Genome Analyzer IIx platform [66], [67]. The average length was also longer than that reported for rubber tree, *Polygonum cuspidate*, *Hypericum perforatum* and safflower transcriptomes obtained using the same platform with this study but different assembly software (SOAPdenovo) from this study (Trinity) except the safflower transcriptome (Trinity) [68]–[71]. The different assembly softwares used probably led to the diversity of average unigene length. The sequence reads produced unigenes (mean = 502.6 bp) even longer than some organisms like *Ziziphus Celata* (mean = 408 bp) and *Salvia miltiorrhiza* (mean = 414 bp) sequenced by Roche 454 GS FLX platform [72], [73].

Quality of the *de novo* assembly was validated by comparison with known databases, functional annotation and marker validation by the PCR amplification. It turned out that a high percentage of unigenes could match to the databases and most of the primers could produce amplicons, indicating the high quality of our assembled unigenes. By far, this is the first report of a large scale of transcriptome sequencing and analysis on celery.

### Functional Annotation and Classification of Unigenes

Due to the lack of a reference genome, it is kind of difficult to estimate the number of genes and predict the potential functions of the transcripts. So we made a BLAST analysis using the public protein databases to indentify genes in our collection indirectly. In our study, 78.28% and 48.93% of the celery transcripts had homologs in the Nr and Swiss-Prot protein database, respectively. The percentage of hits was positive correlation with the number of long sequences, which was confirmed by the fact that 41.05% of unigenes shorter than 200 bp had no BLAST matches while only 1.67% longer than 1,000 bp had no hits. We also observed the same correlation in other studies [18], [64], [65]. The unigenes without hits probably belonged to untranslated regions, non-coding RNA, short sequences not containing a protein domain or assembly mistakes. The results of sequence comparison with Apiaceae ESTs indicated that the unigenes we got might represent potential celery-specific genes, and they would be a great supplement to the current database. 47.02% of unigenes had matches to carrot transcripts, suggesting the possibility of comparative genomics studies.

Large numbers of unigenes were assigned to a great diversity of GO categories (Figure 4) and COG classifications (Figure 5). Most representative transcripts were mapped to specific pathways, such as metabolism, genetic information processing, environmental information processing and cellular processes using the KEGG database. On the other hand, we found unigenes involved in the metabolism of chlorophyll, cellulose and pectin. Orthologs related to bolting and flowering were also detected. These transcripts may be genes controlling specific agronomic traits and they will be useful for gene function study.

By this study, our results demonstrated that high-throughput transcriptome sequencing is an efficient, reliable and inexpensive tool for transcriptome analysis and marker discovery in non-model organisms. The large number of sequences generated in present study will provide foundation for our further studies like marker development and validation, gene mapping and molecular marker-assisted selection breeding.

### EST-SSR Markers Identification

In the previous research on celery, molecular markers were mainly focused on RFLP, RAPD and AFLP markers. Nowadays, polymorphic SSR markers play an important role in genetic diversity research, linkage map construction, identification of varieties, comparative genomics and related analysis. In this study, we performed a general screen on the celery transcripts for the presence of microsatellites and analyzed the distribution and frequency of the markers. A search for repeats from mono- to hexa-nucleotide yielded a total of 2,997 SSRs in 2,601 unigenes, accounting for approximately 6.15% of the total unigenes. This percentage agrees with the range of 4%–11% in previous studies [33], [74].

While as a result of various SSR searching tools and criteria, it’s not surprising that the frequencies, types and distributions of the potential SSRs are sifinificantly distinct in different studies. As was shown in Table 2, di-nucleotide repeats were the most frequent SSR motifs type followed by tri-nucleotide repeats in this study, which was consistent with some organisms reported like sesame [65], rubber tree [68], [75] and red pepper [76] but inconsistent with cereal [77]. The most frequent motif types were greatly diverse in different studies. In this study, GA/TC (29.55%) and TGA/TCA (2.74%) were the most frequent motifs among the di-nucleotide and tri-nucleotide respectively.

### EST-SSR Marker Polymorphism

In present study, a total of 80 primer pairs were designed and used for further assessment of the assembly quality. 65 primer pairs (81.25%) could successfully yield amplicons and 47 primer pairs amplified PCR products at the expected sizes. The others resulted in larger PCR products than expected, which may be due to the presence of introns, a lack of specificity or assembly errors. The failure of 15 primer pairs to produce amplicons may be due to the large introns or insertions, the location of the primer across splice sites, primer synthesis problems or poor sequences quality. Most of the celery EST-SSRs generated high quality amplicons, suggesting that the transcriptome sequencing were accurate and the assembled unigenes were of high quality. Currently there exist only hundreds of genetic markers in celery, so the sequences seem more valuable and can be put to good use in the future. Based on the SSR-containing sequences in our dataset, more PCR primers could be designed and used for germplasm polymorphism assessment, linkage map construction, gene mapping, and comparative genomics in celery.

SSR markers were subjected to polymorphism test and most of the polymorphic SSRs gave no more than 4 alleles, which was in agreement with the previous result by Wang [13]. While the average PIC value in this study (0.35) was lower than that in Wang’s report (0.527) using also SSR markers, which was probably due to a smaller sample size of *A. gravolence* accessions tested here (n = 31) than the previous one (n = 68). However, the average PIC value obtained here (0.35) was higher than that reported by Wang using SRAP markers (0.264), suggesting that SSR is a higher polymorphism marker.

All varieties were classified into two groups at the level of genetic similarity 0.6, which was in good accord with the plant taxonomy based on genetic relationship. Several lines of celery were assigned to a group with celeriac, which may be cause by hybridization between celery and celeriac. Given that local celery and celery may have a similar genetic background and existence of hybridization during the long period of domestication, the result that some local celery lines were scattered to the celery group was nothing out of the ordinary. Molecular markers like SSRs can distinguish varieties without morphological diversities, and they have been a useful tool in research like cultivar classification and genetic diversity analysis.

## Concluding Remarks

In this work, a large EST dataset composed of 42,280 transcripts was achieved. The functional annotation and classification were beneficial for us to have a better understanding of celery genomic information. Based on the sequences, 2,997 SSRs were identified and characterized as potential molecular markers. 80 primer pairs were randomly selected to detect polymorphism among 31 celery accessions, and 65 pairs (81.25%) successfully amplified fragments with 27 pairs showing polymorphic loci.

Through this research, we obtained a large-scale transcriptome dataset using relatively short reads generated by paired-sequencing technology. This was the first try on celery, an organism lacking genomic resources. The dataset will be important for gene discovery in celery and for annotation of the celery genome. The indentified unigenes involved in specific pathways provided candidates for genes controlling petiole traits, bolting and flowering. The SSRs indentified here will supply an important resource for constructing genetic linkage maps, mapping interested genes and marker-assisted breeding in celery. On the other hand, celery along with carrot, coriander, and fennel belong to umbelliferous vegetables. So the resource will enhance comparative genomics studies within the family and plants related.

## Supporting Information

Table S1 **Primer pairs validated in this study.**
(XLS)

Table S2 ***Apium graveolens*** **L. germplasms for polymorphism validation with EST-SSRs.**
(XLS)

Table S3 **Summary of ORF-containing unigenes.** Unigene length and predicted position of ORFs were indicated for the whole unigene collection.
(XLS)

Table S4 **The main identified chlorophyll metabolism-related genes potentially controlling petiole color.**
(XLS)

Table S5 **The identified genes involved in cellulose and pectin metabolism.**
(XLS)

Table S6 **Genes potentially controlling bolting and flowering.**
(XLS)
